# The Predisposing Causes of Dental Caries

**Published:** 1873-05

**Authors:** Jerome B. Ten Eyck


### ARTICLE III.

### *Predisposing Causes of Dental Caries.*

BY JEROME B. TEN EYCK, M. D., D. D. S.

In discussing the subject of dental caries, our remarks will be confined principally to those predisposing causes dependent upon *defective nutrition.*

Investigations have proven to us that the teeth of past generations have not been found to be attacked with caries to so great an extent, by far, as those in modern times ; and that in countries the most advanced in civilization, there do we find that dental caries prevail to the greatest extent.

That there are many causes predisposing to dental caries, in connection with, and independent of defective nutrition, is beyond peradventure. We observe in that peculiar constitutional habit of the body called *diathesis*, which is entailed from one generation to another, the causes of various pathological conditions manifested in the entire human organism, but nowhere more distinctly marked than in the dental structures. In the temperaments which are characterized by various constitutional tendencies, and which are amenable to laws of nature that are fixed and certain, do we find the physical character, and pathological condition of the dental organs in different types.

Some medicines have been proven to have various deleterious tendencies upon the constitution, and directly or indirectly to influence the vitality of the teeth. Infectious diseases, as well as epidemic and others, sow some of the seeds of premature decay. The effects of impure air, such as poisonous exhalations from swamps, and decomposing filth from cities, are among the causes of defective development.

The predisposing causes above referred to, with many others, which we omit to mention, operate to a greater or less extent in every country, even where dental decay is rarely seen. It is evident, therefore, that there is some cause incident to the mode of life of the people in civilized countries, paramount to the above mentioned causes, which exercises a deleterious and destructive influence upon the dental organs.

The science of physiology teaches us, that the formative material which developes bone, muscles, tissues, and every part of the human organism, is produced from the blood; and that the blood is formed from the food ingested and assimilated in the system. It is evident that the blood to be pure and good, must require *pabulum* containing all the necessary ingredients to form the different tissues of the body, or imperfect development must inevitably ensue.

Civilization with its artificial methods of preparing, destroying, and depriving food of its proper nutritive elements, is responsible to a great extent for the lack of development so alarmingly manifested in the early decay of teeth. Chemistry teaches us that *lime salts* not only constitute the principal part of the bony structure, but that they enter into the composition of every other tissue of the body. The daily quantity which is necessary to maintain the body in health, bring for the bony tissues, about one per cent. of their weight, exclusive of what is necessary for other tissues of the body. This would seem a large proportion of the mineral elements required for the *removal* of waste material of osseous tissue; but much more is required, we may reasonably infer, to daily *build* the tissues during the period of growth.

The greatest mischief ensues to the dental organs when the mineral elements are denied to the embryo and infant, for it is during these periods that the teeth are developed and formed, which, if defective, nature can never restore; consequently if the mother does not replenish her blood with the required constituents, the teeth of the child must inevitably and irretrievably suffer. It is a well known fact that women, particularly of our civilized country, make fine flour of wheat the principal portion of their subsistence, and the same remark applies to children for the first seven or eight years of their lives; the consequence, as regards the teeth, is that their tissues are starved, the dental tubules are not normally calcified, neither is the enamel hardened with the flinty quality which it ought to have, excepting perhaps on its surface, which is superficial and does not extend the whole length of the enamel columns. The dental surgeons find these cutting easily with their excavators and chisels. The lines on the enamel of these teeth making the boundaries of the different circles of calcification are deep, extending to the dentine in many instances, and affording a lodgement for particles of food or other extraneous substances capable of fermentation and forming acids, which together with the acid secretions of the mouth when morbid, dissolve the lime salts from the dental tissues. The vital process having been interfered with in its microscopical structure, the cells are broken down by a low grade of inflammation, and the cavity of decay grows larger day by day.

If the mother does not ingest sufficient mineral substance in her food to nourish and build up the bones and teeth of her child, whether "in utero" or at the breast, a portion of her own supplies will be extracted from her osseous tissues for this purpose; and here may be assigned one of the principal reasons why mothers suffer so much from dental decay and tooth-ache.

That salts are furnished the child which are abstracted from the mother's tissues, is said to be proven by the greatly diminished amount of effete salts excreted by a nursing mother who is not receiving ingesta in sufficient quantity. Effete lime salts are daily cast out of the body, chiefly in the urine, by those even undergoing the process of starvation. The retention of them would seem to be demanded if consistent with physiological harmony, but nothing short of the demands of a new being *seems* to command their retention or conversion into vitalized factor of nutrition.

Although most of the articles of food, fruits vegetables, meats, &c., are deprived of their mineral elements by processes of cooking, *that* article acknowledged in all countries to be the staff of life, and which should be kept inviolate from the mischievous devices of man, is the one of all others the most completely robbed of its important nutritive properties.

The white and glistening starch cells constitute the principal bulk of *fine* flour of all grains, and these cells are very poor in phosphates. The gluten cells are the great magazines of the phosphate of lime; the gluten cells are dark colored, and in wheat lie in a single layer directly beneath the bran coat. To the bran they mostly adhere when wheat is ground for *white flour.* Prof. Hosford of Cambridge, in his pamphlet on "bread making," shows some cuts of gluten cells adhering to commercial bran, as seen under the microscope. The experiments of Johnston, of England, and Mayer of Germany, have found *fifteen times* more of the phosphates in *unbolted wheat*, than in superfine flour, proving that the bolting process abstracts almost entirely the elements demanded for the nutrition of the osseous tissues.

The Creator in his wisdom has provided all the necessary elements of nutrition in suitable proportions in every kind of food which would seem to be natural to man, in whatever clime he may choose for his habitation. As the phosphates of lime are so imperatively demanded by all living creatures, so also are they found in abundance in every kind of food in its natural condition, whether corals, legumens, roots, or the flesh of animals.

As the dental organs of the present generation are inferior in durability to those of the preceding one, so if the same causes continue to exist, added to those acquired by inheritance, the injury *must* increase with each succeeding generation, until we at last, reasoning from analogy, become a toothless people.

The duty of the dental and medical professions do not end with the cure or repair of disease; it is a higher and more imperative one to prevent it; the people should be instructed in the laws of health, and those mighty evils which are being perpetuated and increased, should be proclaimed all over the land, until the people are made to see the alarming evils of their ways.

